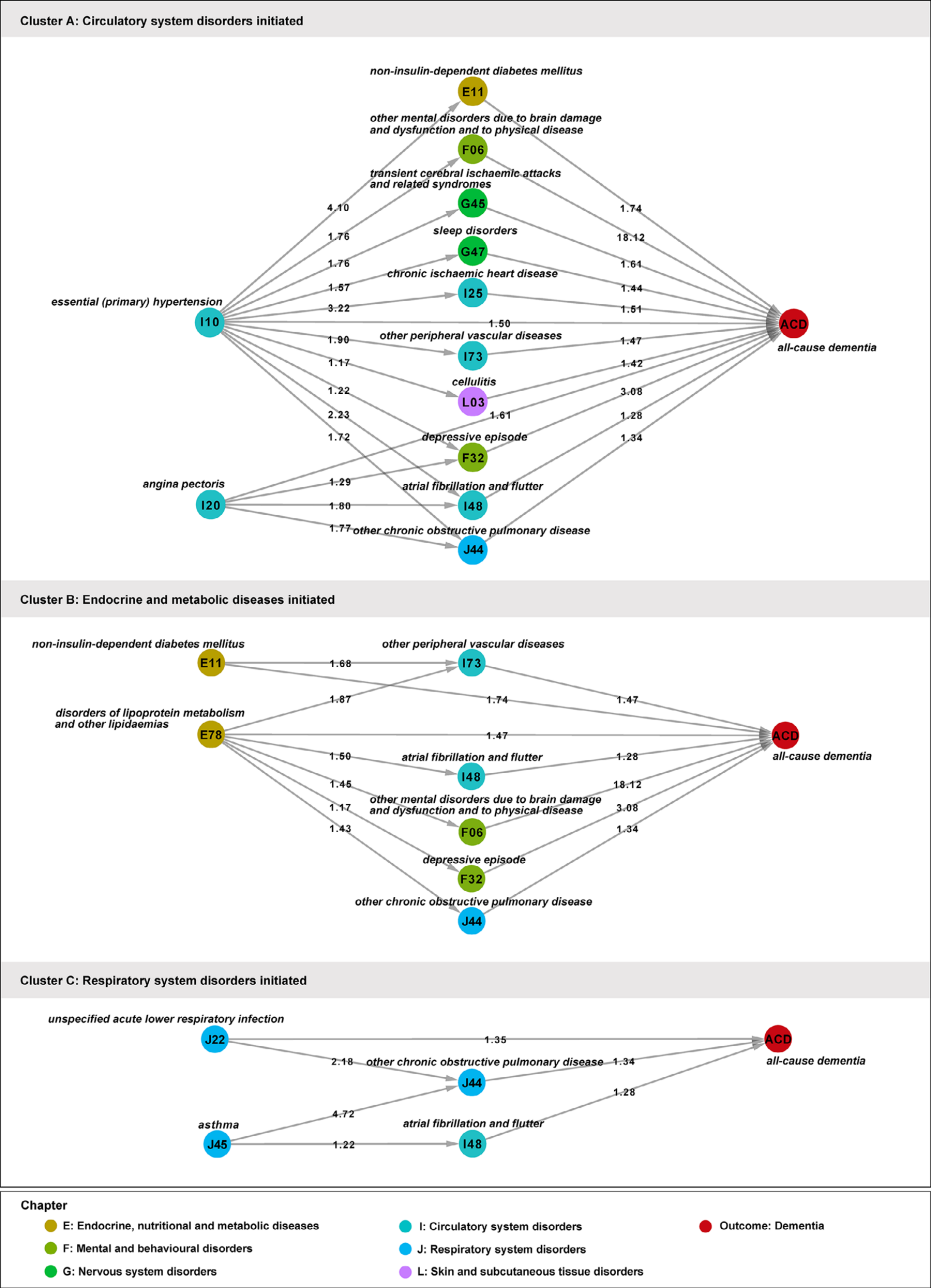# Disease trajectories before dementia: Evidence from a large‐scale community‐based prospective study

**DOI:** 10.1002/alz.095010

**Published:** 2025-01-09

**Authors:** Jialin Li, Ding Xia, Mei Cui, Yingzhe Wang, Jincheng Li, Li Jin, Xingdong Chen, Chen Suo, Yanfeng Jiang

**Affiliations:** ^1^ State Key Laboratory of Genetic Engineering, Human Phenome Institute, Zhangjiang Fudan International Innovation Center, Fudan University, Shanghai, Shanghai China; ^2^ Fudan University Taizhou Institute of Health Sciences, Taizhou, Jiangsu China; ^3^ Ministry of Education Key Laboratory of Public Health Safety, School of Public Health, Fudan University, Shanghai, Shanghai China; ^4^ Huashan Hospital, Fudan University, Shanghai, Shanghai China

## Abstract

**Background:**

Dementia patients often have several co‐existing diseases, whereas the specific temporality and development patterns between them remain uncertain.

**Method:**

Based on the multicenter, community‐based prospective UK Biobank, enriched disease diagnoses were extracted from hospital admission linkage, along with basic demographic information and individual genome data from the baseline visit. We constructed disease trajectory before dementia utilizing the phenome‐wide association analysis to firstly filter potential precursors, the directional test to select temporal disease pairs, and conditional logistic regression to finally quantify association strength. Disease trajectory was also constructed across diverse subpopulations stratified by dementia subtypes (Alzheimer’s disease and vascular dementia), sex, median diagnosis age, and *Apolipoprotein E* (*ApoE*) ε4 status (carrier or not), respectively. Mendelian randomization (MR) was further adopted to suggest causality for longitudinal associations.

**Result:**

Our study comprised 434,266 participants without baseline dementia and 4,638 individuals with all‐cause dementia, with a median follow‐up of 10.9 years. Initially, 1,253 diseases were extracted as potential nodes of disease trajectory preceding dementia. We ultimately defined three clusters of disease trajectory preceding all‐cause dementia, initiated by circulatory, metabolic, and respiratory system diseases (Figure 1). The initial diseases in the trajectory were diagnosed approximately 5‐15 years before dementia. Cerebral infarction or chronic renal failure following chronic ischemic heart disease was the specific trajectory before vascular dementia. Angina, progressing to atrial fibrillation and then dementia, is a key trajectory in males, senior dementia patients, and *ApoE* ε4 non‐carriers. Dementia patients without *ApoE* ε4 typically developed urinary system disorders after musculoskeletal and connective tissue diseases. Lipid metabolism disorders remained in trajectories across all subgroups. However, MR based on inverse variance weighting indicated a modest causal relationship between lipid disorder and Alzheimer’s disease (Odds ratio = 1.05, *P* value = 0.234).

**Conclusion:**

This study offers a comprehensive view of longitudinal disease trajectories before dementia among diverse subpopulations, indicating critical precursor diseases and specific development patterns. The findings emphasize the importance of clinical attention to patients with cardiometabolic dysfunction in midlife for screening and prevention of dementia. Further population studies and mechanism explorations are needed to validate and generalize the trajectories.